# Financial constraints, government subsidies, and corporate innovation

**DOI:** 10.1371/journal.pone.0259642

**Published:** 2021-11-10

**Authors:** Qi Li, Jiaojiao Wang, Guohua Cao, Jing Zhang

**Affiliations:** 1 School of Finance, Chongqing Technology and Business University, Chongqing, China; 2 School of Economics and Administration Business, Chongqing University, Chongqing, China; Universitat de Valencia, SPAIN

## Abstract

To investigate the relationships between financial constraints, government subsidies, and corporate innovation, a semi-logarithmic fixed-effect panel model and mediation effect test were applied, based on the data of Chinese listed companies from 2007 to 2017. We find that (1) financial constraints suppress corporate innovation. (2) Government subsidies are targeted at bailing out firms facing financial constraints. (3) Government subsidies promote corporate innovation (4) Government subsidies partially offset the suppression of financial constraints on innovation. We contribute to the fields of public finance, corporate finance, and corporate innovation by: (1) justifying the government subsidies target strategy as a bailout of corporate financial constraints, (2) verifying the corporate-innovation promotion of government subsidies, thus justifying the efficiency of government subsidies, and (3) showing that different types of innovation benefit differently from subsidies, thus justifying subsidies as a structural innovation engine.

## 1 Introduction

Chinese corporations have been accused of benefiting from potential unfair competition caused by the government subsidies; this has drawn more attention since the Huawei-related events. Considering the wide application of government subsidies worldwide, this debate points to a major issue in policy making, namely the target of government subsidies, which has been discussed for years [1–3]. One of the most accepted targets of government subsidies is “picking the winner” [1], a strategy for subsidizing innovative corporations. However, the picking-the-winner strategy with a large subsidy amount might cause unfair competition. Another influential target is the political connection bias in the information-asymmetry context [2, 4, 5]. This strategy might cause corruption, and thus lead to the under-efficiency of subsidies [4, 6].

However, in China, the existing research neglects the fact that governmental support is intended to alleviate corporate financial constraints [7, 8], even though this endogeneity has often been implied before [9, 10]. The core values of Chinese socialism should be considered, which require government subsidies to “support small and medium-sized enterprises” [11] and to “alleviate the difficulties of market entities and maintain necessary policy support for achieving this goal” [12]. Therefore, China should not be accused of unfair competition because China’s goal is to use government subsidies to bail Chinese corporations out of their financial constraints [8]. Meanwhile, the bailing-out target of government subsidies might incidentally promote innovation; thus, the potential under-efficiency caused by corruption should not be a concern. According to market competition theory, financial constraints promote corporate innovation because corporations tend to pursue competitive advantages by innovating [13]. Moreover, whether government subsidies promote or crowd out corporate innovation is still being debated in recent research [14]. Another concern is the possible bias or corruption related to political connections [4, 6].

To determine the allocation process and innovation-promotion process of government subsidies, we empirically tested the data of Chinese listed companies from 2007 to 2017. To determine the mechanism, we constructed a semi-logarithmic fixed-effect panel model, mediation effect test, and moderation test. As for the potential targets of government subsidies, we introduced innovative abilities, political connections, and financial constraints, and for the efficiency of government subsidies, we considered corporate innovation.

By doing so, we found the following: (1) financial constraints suppress corporate innovation. (2) Financial constraints are the targets of government subsidies, while no evidence of innovation ability or political connection is found. (3) Government subsidies promote corporate innovation. (4) Government subsidies partially and competitively mediate the suppression of financial constraints on corporate innovation. (5) There is a structural difference between exploratory and exploitative innovation.

Based on the results, we contribute to the fields of public finance, corporate finance, and corporate innovation. First, we examine the target of government subsidies as corporate bailouts, and verify the government subsidies aimed at alleviating financial constraints. Second, we verify whether financial constraints suppress corporate innovation in the Chinese context. Finally, we verify the efficiency of government subsidies from the perspective of corporate innovation.

The contents are as follows: Section 2 reviews related literature and presents hypotheses; Section 3 selects variables and sets up empirical models; Section 4 estimates the models and analysis results; Section 5 examines subsamples and introduces different estimating settings as robustness tests; and Section 6 presents our conclusions.

## 2 Literature review and hypotheses

### 2.1 Targets of government subsidies

What is the target of government subsidies? Promoting innovation has become a critical developing target for a nation [15]. Thus, fostering corporate innovation through government subsidies has become a common practice globally [7, 16–19]. From the perspective of optimizing resource allocation, subsidizing the most successfully innovative corporations is the best choice. Thus, picking-the-winner is the most widely accepted targeting strategy of government subsidies [1]. The goal of picking the winner is to subsidize innovative corporations. The rationale behind the picking-the-winner strategy is the risk of innovation and information asymmetry, which together generate a market failure of underinvestment in innovation. Thus, government subsidies are widely accepted to fill the market failure gap [20].

Given that the advantage of cheap labor is gradually disappearing, the importance of innovation is becoming more urgent in China [4]. Thus, the picking-the-winner strategy seems to suit China, where there is an urgent need to support innovation by subsidizing corporations with superior innovative ability.

Meanwhile, considering the information-asymmetry condition, it is also argued that government subsidies allocations might be biased by political connections, because political connections provide channels for information [2, 4, 5]. By sharing information between governments and corporations [5], political connections provide corporations with economic benefits, such as access to or opportunities for subsidies [4]. However, this strategy might cause corruption, and thus lead to the under-efficiency of subsidies [4, 6]. For example, it is argued that governments contribute more to corporations with better financing in Poland [21]. Thus, many studies indicate distortion of resource allocation by public bureaucracies [9], and argue that the political resource curse effect and crowding out effect may hinder corporate innovation and its performance [6]. Concerns also loom that local governments are more inclined to provide opportunities to companies that have good relationships with them, resulting in distortions in the allocation of government resources [22]. Although the Chinese government has tried to phase out its role as a central planner, it still plays a considerable role in the national economy, and political connections tend to bring corporations various benefits, including subsidies [23].

However, considering the core values of Chinese socialism, there might be a new target of Chinese government subsidies. In other words, Chinese government subsidies aim to bail Chinese corporations out of their financial constraints [8]. Several developing reports from the Chinese central government indicate the requirement of government subsidies to “support small and medium-sized enterprises” [11] and to “alleviate the difficulties of market entities and maintain necessary policy support for achieving this goal” [12]. Besides the appealing, funds have been made available to support small and medium-sized enterprises in China [24]. Thus, Chinese government subsidies might not be accused of unfair competition. Meanwhile, the bailing-out target of government subsidies might incidentally promote innovation, and so the potential of under-efficiency caused by corruption should not be a concern. A similar argument has been empirically proven in Colombia [19], while little research has been done in China, to the extent of the authors’ knowledge. Thus, the following hypothesis is proposed:

**Hypothesis 1 (H1):** In China, corporations with more severe financial constraints receive more government subsidies.

### 2.2 Corporate innovation and effectiveness of government subsidies

It is widely accepted that government subsidies promote corporate investment [10, 20, 25], mainly by filling the gaps between private investment and optimal levels, where gaps are caused by market failures such as information asymmetry [17]. In transition economies, subsidies are the most direct means for the government to support enterprises as an “invisible hand” [26].

However, even though economic theory justifies government subsidies for alleviating market failures, it also points out concerns about its effectiveness and efficiency [9]. Classical economic theory is particularly concerned with the effectiveness of governments in promoting corporate innovation [14]. Governmental intervention is recommended to be indirect (e.g., by creating a preferable environment) instead of taking a “hands-on approach” [27], because direct subsidies might generate a possible crowding-out effect [7, 13]. Researchers also concern that the attached restrictions of government subsidies might hinder innovation, for instance, through prohibitions on transferring knowledge away from a given location [28]. Another concern is the problem of corruption, which leads to under-efficiency [4, 6]. Thus, whether subsidies promote innovation remains an unsettled empirical question [14, 29].

We argue that government subsidies promote corporate innovation. The fundamental rationale is that innovations technically require additional resources, and subsidies fill the gap [23]. The innovation-promoting effect of government subsidies comes from innovation incentives and risk-taking preferences [17], and improves the prospects of innovation [30]. Innovation is a kind of public good, namely through non-rivalry and non-excludability [18]. Thus, innovation provided by the private sector is less than the optimal level [9, 31]. Technology-driven corporations must fund research to develop new products or services [7], and subsidies offset the financial constraints caused by market failures by compensating for innovation input [9]. Moreover, government subsidies with specific requirements (e.g., R&D taxes) hedge uncertainty in innovation and spur innovating enterprises to acquire continuous subsidies [32]. Specifically, targeted government subsidies help to reallocate labor [33], and more talented human capital promotes corporate innovation in addition to funding support [34]. Furthermore, a more innovative atmosphere leads to a more positive external environment for collaboration [35], and this helps knowledge spillover among technology-driven corporations.

There is practical evidence supporting our argument that corporations receiving governmental support perform better in innovation [36]. For example, innovative activities are promoted by favorable loan policies in Spain [37]. In China, government subsidies for innovation are an important means for the government to adjust the allocation of innovation resources and guide corporations’ R&D activities. In China, a positive relationship between innovation investment and subsidies has been found [38]. Chinese researchers such as Lu and Guan (2020) found evidence that government subsidies both directly and indirectly increase the available funds, set off R&D costs, and alleviate financial constraints [39]. Thus, the following hypothesis is proposed:

**Hypothesis 2 (H2):** In China, corporations with more government subsidies generate more innovation outcomes.

### 2.3 Target and effectiveness of government subsidies

It is widely accepted that financial constraints hinder corporate innovation [20], and some researchers consider financial constraints as the major obstacle [18].

From the resource-based view, resource dependence theory explains this as follows: corporate innovation is highly resource-dependent, R&D is time-consuming, and the process is uncertain. For most corporations, R&D investment cannot be funded by internal financing. However, the need to obtain external financing is difficult as well, due to the asymmetry of information and the uncertainty of external resource acquisition. Thus, financial constraints have arisen. As a result, severely financially constrained corporations might have to abandon prospective R&D projects; thus, both corporations’ incentives to innovate and countries’ innovative investments are damaged.

However, from a market-competition perspective, it is argued that the lack of access to financial resources is what encourages corporations to pursue market innovations [40]. These counter-intuitive events have been observed in developing countries such as China [41] and Vietnam [42]. The reason behind this might be the market-competition hypothesis, especially in the Chinese context: world-spotlight economic development energizes investments in innovation. To grasp the future competitive advantages, given such a huge niche market in China, innovative incentives might even break the hindrance of financial constraints. Thus, the following hypothesis is proposed:

**Hypothesis 3 (H3):** In China, corporations with more severe financial constraints generate more corporate innovation outcomes.

### 2.4 Mediation of government subsidies

The mediation of government subsidies has two components: the one is that corporations with more severe financial constraints receive more government subsidies, the other is that these subsidized corporations generate more innovation outcomes.

Considering the core values of Chinese socialism, the Chinese government subsidies aim to bail Chinese corporations out of their financial constraints. To bail out corporations has shown in several reports of the Chinese central government [11, 12], and funds have been prepared [24]. Thus, H1 states that government subsidies target financial constraints: corporations with more severe financial constraints receive more government subsidies. This summarizes the first component of the mediation of government subsidies.

When companies face financial constraints, government subsidies reduce the cost of corporate R&D, thus promoting their willingness to innovate. Due to the imperfect intellectual property system and distortion of the market, corporations may lack sufficient internal motivation for innovation to meet the socially optimal level. A government’s policy of subsidizing corporate innovation activities can effectively encourage corporations to increase their investment in innovation. Based on signal transmission theory, corporations that receive government innovation subsidies convert the behavior of receiving subsidies into a signal mechanism that reflects their own capabilities for innovation and competitive advantages in the market. This mechanism provides an invisible guarantee for corporations, making it easier to obtain external financing and ease credit constraints. Meanwhile, innovation competitiveness supported by government subsidies will increase the attractiveness of corporations to high-end talents, thereby promoting innovation performance. Thus, H2 states that government subsidies promote corporate innovation, namely corporations with more government subsidies generate more innovation outcomes. This summarizes the second component of the mediation of government subsidies.

Thus, taking the two components mentioned above, the hypothesis of government-subsidy mediation is as follows:

**Hypothesis 4 (H4):** Government subsidies mediate the relationship between financial constraints and corporate innovation.

### 2.5 Moderation of government subsidies

Previous research implies that the way government subsidies interact with financial constraints influences R&D investment [14, 20], which indicates a moderating effect between government subsidies and financial constraints on corporate innovation [43]. Takalo and Tanayama (2013) model the interactions between subsidies and corporate financial conditions, and argue that the expected effects of subsidies are heterogeneous; that is the optimal subsidy level depends on the severity of financial constraints [44]. Brzozowski and Cucculelli (2016) suggest a better innovation-promotion effect of government support that facilitates external funding, indicating the interaction between subsidies and financial constraints [45].

The mechanism behind this moderation might be threefold. The first is the diminishing effect of financial capital. In other words, once a corporation receives both public subsidies and private funds, even if all capital is invested in innovation, the return margins might diminish. The second is the crowding-out effect of government subsidies. That is, some funds might be diverted to other uses. The final one is moral hazard. Managers might seek private profits and shareholders might strengthen governance even at the risk of lower efficiency. In China, managers tend to gain private profits more easily when receiving larger government subsidies [46]. Meanwhile, imperfect corporate governance provides opportunities to embezzle subsidies. Thus, based on the above analysis, we propose the following:

**Hypothesis 5 (H5):** Government subsidies positively moderate the relationship between financial constraints and corporate innovation.

**Fig 1:**
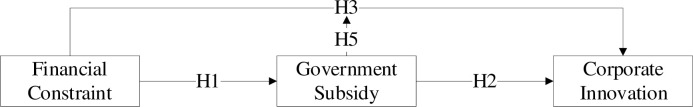
Theoretical framework.

The framework constructed by the hypotheses above is shown in Fig 1.

## 3 Research design

### 3.1 Variable selection

#### 3.1.1 Dependent variable: Corporate innovation

In contrast to Carboni (2017) who measures innovation by R&D expenditure [17], we apply the patent approach according to mainstream Chinese research [47–49]. The rationale of the patent approach is threefold: First, patents reveal the outcome of innovation and thus show innovation efficiency, while expenditures indicate more about the propensity for innovation. Second, patents granted by governments are a more credible measure than expenditure information in accounting reports of corporations. Third, measuring innovation using patents as output is of high interest to Chinese policymakers [50].

Corporate innovation is measured by *ln*(*Application Number of Patent* +1), denoted as *ln Apply*_*it*_. Specifically, Chinese patent law divides patents into the categories of invention, utility, and design. The number of patent applications used in this study is the sum of these three types. The different types were also separately tested in the subsample part as robustness tests.

#### 3.1.2 Independent variable: Financial constraint

There are three mainstream measures of financial constraints in existing studies: investment-cash flow sensitivity [51], KZ index [48], and SA index [47, 49]. Among them, the investment-cash flow sensitivity and KZ index are constructed using multiple endogenous variables (such as cash flow), and are thus subject to the availability and authenticity of data. Conversely, the variables used in the measurement of the SA index are exogenous, alleviating possible endogenous problems. Meanwhile, the SA index is only related to the size and age of the company, which has been proven to be a measure of financial constraints under various circumstances. Therefore, following mainstream research in China [47, 49], the SA index is applied to measure financial constraints, denoted as *SA*_*it*_. The calculation of *SA*_*it*_ is as follows:

$${SA}_{it}=0.043\;\ln^{2}\;Asset_{it}-0.737\;\ln\;Asset_{it}-0.040ListDuration_{it}$$

where *Asset*_*it*_ denotes the scale of assets, and *ListDuration*_*it*_ denotes the duration of corporate listing. *SA*_*it*_ is a negative indicator, namely a larger *SA*_*it*_ indicates a less severe financial constraint. Considering the existing research arguing a non-linear influence of financial constraints [14, 20, 43], a squared form of the SA index is introduced, denoted as ${SAsq}_{it}={SA}_{it}^{2}$.

#### 3.1.3 Mediator: Government subsidies

Following mainstream research in China [10, 38], the government subsidy is denoted as *lnPltcSbsd*_*it*_ = *ln*(*Subsidy received by Corporation* +1). In contrast to early research that simply measured subsidy with a dummy variable [52–55], a continuous measure is applied to include more information. The logarithm form is introduced to avoid distribution skewness and to alleviate problems caused by dimensional differences.

#### 3.1.4 Control variables

Six dimensions of the control variables are considered here. The first of these is the potential targets of government subsidies. Political connections are considered and denoted as *PltcCnct*_*it*_, according to recent research suggesting that political connections lead to economic benefits, including government subsidies [56, 57]. Innovation capacity is considered according to the picking-the-winner subsidizing strategy [1], denoted as *ApplyPerNtCpt*_*it*_ and *ApplyPerLbSlr*_*it*_, where the former indicates the average innovation productivity of capital and the latter indicates that of employees.

Second, we examine the corporate characteristics. The duration of establishment is considered, denoted as *EstbDrtn*_*it*_, because older corporations may have more capital, superior technology, and an established reputation [7, 16, 17, 38]. Along with the similar rationality, the duration of listing is also considered according to Lin and Liu (2017) [58] and Su and Xiao (2019) [5], denoted as *ListDrtn*_*it*_. Following Conti (2018) [28], the amount of shareholding is considered to be *SHSize*_*it*_. Whether corporations are owned by the state is considered because there are concerns that state-owned enterprises obtain more resources [10, 58], denoted as the dummy variable *SOE*_*it*_. Following Liu and Li (2016) [10] and Busom and Vélez-Ospina (2017) [19], whether corporations owned by entities outside China are considered, denoted as dummy variable *FOE*_*it*_; the ratio of foreign shareholdings is also considered, denoted as *ForeignOwn*_*it*_. The ratio of institutional shareholdings is also considered following Su and Xiao (2019) [5], denoted as *InstOwn*_*it*_.

The third dimension is corporate finance. The asset scale is considered, measured as a logarithmic asset and denoted as *ln Asset*_*it*_, because larger assets help in securing mortgages and indicate that there is less risk than there would be with smaller competitors [7, 16]. Leverage is also considered, measured by the debt-on-asset ratio and denoted as *Leverage*_*it*_, because high leverage implies more debt, which undermines solvency [16]. Profit is measured by the return-on-asset ratio, denoted as *ROA*_*it*_, because revenue indicates how much a corporation earns, which is the primary indicator of solvency [7, 16]. Growth is also included [58, 59], denoted as *RevGrowth*_*it*_.

Fourth, we examine corporate governance, following Wang and Li (2019) [60]. This includes the number of major shareholders connected, denoted as the category variable *SHCnnct*_*it*_. Whether the CEO and chair of the board are the same person is denoted as the dummy variable *Duality*_*it*_. The number of directors is denoted as *DBSize*_*it*_, the number of director board meetings as *DBMeet*_*it*_, the number of supervisors as *BSize*_*it*_, the number of supervisor board meetings as *SBMeet*_*it*_, and the number of auditors as *AuditNo*_*it*_. Whether the auditing institution is one of the Big Four is denoted as dummy variable *BigFour*_*it*_, while whether it is an entity outside China is denoted as *ForeignAudit*_*it*_.

Fifth, we examine the characteristics of the CEO and chair of the board, mainly according to the upper echelon theory. The CEO’s age is denoted as *CEOAge*_*it*_, their education as *CEOEdct*_*it*_, and their gender as dummy variables *CEOMale*_*it*_. The chairman’s age is denoted as *DrctAge*_*it*_, their education as *DrctEdct*_*it*_, and their gender as *DrctMale*_*it*_.

Finally, we examine the financial markets. The earnings per share are denoted as *EPS*_*it*_. Tobin’s Q value is included following Lv and Zeng (2018) [61] and Su and Xiao (2019) [5], as *TobinQ*_*it*_. The book-to-market ratio is denoted as *BM*_*it*_, turnover of tradable stock is denoted as *Turnover*_*it*_, and volatility of stock price is denoted as *Volatility*_*it*_.

Following Bloom and Schankerman (2013) [31], Grilli and Murtinu (2014) [27], and Mateut (2018) [20], the fixed effects of individuals, time, and industry are considered. The model is set as a fixed model, and dummy variables for each year (denoted as *yr*_*it*_) and industry (denoted as *ind*_*it*_) are included.

### 3.2 Data source

Chinese A-share listed corporations were chosen as the sample and the time span is from 2007 to 2017; these were chosen primarily because of data accessibility and authenticity. Data were collected from China Stock Market & Accounting Research (CSMAR) database and China Economic and Financial Research (CCER) database. Missing data are either manually substituted according to annual reports or deleted if the gaps cannot be filled. Observations with obvious mistakes were either manually corrected or deleted if they could not be corrected. Data are winsorized according to the common process: of a certain variable, the abnormal values smaller (or larger) than the 1st (or 99th) percentile is replaced by the 1st (or 99th) percentile of this variable, following the equations below:

$$\left\{ \begin{array}{c} obs_{it}=A,\;\forall \;obs_{it}<A,where\;A\;denotes\;the\;1st\;percentile\\ obs_{it}=B,\;\forall \;obs_{it}>B,where\;B\;denotes\;the\;99th\;percentile \end{array}\right.$$

After data filling, correction, and winsorizing, 14,825 year-corporation observations remained. The results are summarized in Table 1. The correlation matrix is shown in Table 2.

**Table 1:** Summary of statistics.

	N	Mean	Std. Dev.	Min	Max
*lnApply*_*it*_	14825	3.0198	1.4043	0.6931	6.7845
*ApplyPerNtCpt*_*it*_	14825	0.1102	1.1070	0	103.8869
*ApplyPerLbSlr*_*it*_	14825	0.1893	0.3850	0.0001	23.2413
*SA*_*it*_	14825	4.2763	1.5910	0.5248	10.6046
*lnPltcSbsd*_*it*_	14825	16.0394	2.6227	0	20.2369
*PltcCnct*_*it*_	14825	0.3011	0.4588	0	1
*lnAsset*_*it*_	14825	22.1786	1.3215	18.9272	26.5378
*Leverage*_*it*_	14825	0.4527	0.2090	0.0508	1.3447
*ROA*_*it*_	14825	0.0403	0.0580	-0.3098	0.2309
*RevGrowth*_*it*_	14825	0.1813	0.4851	-0.6885	4.271
*EstbDrtn*_*it*_	14825	15.1120	5.6700	0	50
*ListDrtn*_*it*_	14825	9.3864	6.4767	0	27
*SHSize*_*it*_	14825	57577.0060	67783.2160	0	391856
*SHCnnct*_*it*_	14825	2.0541	0.9443	0	3
*InstOwn*_*it*_	14825	4.0213	7.5204	0	75.4950
*ForeignOwn*_*it*_	14825	0.0831	0.8728	0	52.5000
*SOE*_*it*_	14825	0.2745	0.4463	0	1
*FOE*_*it*_	14825	0.0241	0.1533	0	1
*Duality*_*it*_	14825	1.7367	0.4774	0	2
*DBSize*_*it*_	14825	8.8707	1.8621	0	15
*DBMeet*_*it*_	14825	5.1734	5.1329	0	21
*SBSize*_*it*_	14825	3.7369	1.2054	2	9
*SBMeet*_*it*_	14825	1.6461	2.5879	0	10
*AuditNo*_*it*_	14825	1.1381	1.0142	0	3
*BigFour*_*it*_	14825	0.0385	0.1924	0	1
*ForeignAudit*_*it*_	14825	0.0205	0.1417	0	1
*DrctAge*_*it*_	14825	52.2690	8.5237	0	85
*DrctEdct*_*it*_	14825	2.6971	1.6183	0	6
*DrctMale*_*it*_	14825	0.9503	0.2174	0	1
*CEOAge*_*it*_	14825	48.0837	8.5214	0	80
*CEOEdct*_*it*_	14825	2.6881	1.6010	0	6
*CEOMale*_*it*_	14825	0.9354	0.2457	0	1
*EPS*_*it*_	14825	0.3921	0.5253	-1.4076	2.4621
*TobinQ*_*it*_	14825	1.9833	1.2556	0	9.1090
*BM*_*it*_	14825	0.5919	0.2476	0	1.1010
*Turnover*_*it*_	14825	0.0159	0.0244	0	0.1691
*Volatility*_*it*_	14825	0.0173	0.0165	0	0.0582

**Table 2:** Correlation matrix of main variables.

	*lnApply*_*it*_	*SA*_*it*_	*lnPltcSbsd*_*it*_	*PltcCnct*_*it*_	*ApplyPerNtCpt*_*it*_	*ApplyPerLbSlr*_*it*_
*lnApply*_*it*_	1					
*SA*_*it*_	0.4113*	1				
*lnPltcSbsd*_*it*_	0.3844*	0.5322*	1			
*PltcCnct*_*it*_	0.0070	0.0467*	0.0715*	1		
*ApplyPerNtCpt*_*it*_	0.1001*	-0.0489*	-0.0152	-0.0073	1	
*ApplyPerLbSlr*_*it*_	0.3370*	-0.1511*	-0.0569*	0.0305*	0.2844*	1

Note

* denote significance at the 10% significance levels.

### 3.3 Empirical model and estimation method

#### 3.3.1 Baseline model and estimation method

Based on the panel constructed by the corporation-year observations, a semi-logarithmic model with fixed effects was constructed as model (1). To alleviate the problem of endogeneity, the independent and control variables were lagged by one year.

$$\ln\;Apply_{it}=\alpha_{0}+\alpha_{1}SA_{it}+\alpha_{2}lnPltcSbsd_{it}+A_{1}Control_{it}+A_{2}{FE}_{it}+\varepsilon_{it}$$ (1)

where *i* and *t* denote the corporation and time, respectively *ln Apply*_*it*_ denotes corporate innovation, *SA*_*it*_ denotes financial constraint, *lnPltcSbsd*_*it*_ denotes government subsidy, ***Control***_***it***_ denotes the vector of control variables. ***FE***_***it***_ denotes the vector of fixed effects, which includes corporation (denoted as *corporation*_*i*_, and only varies with individual), time (denoted as *year*_*t*_, and only varies with time), and industry (denoted as *industry*_*it*_, and varies with individual and time). *ε*_*it*_ denotes residual. *α*_0_, *α*_1_, *α*_2_, ***A***_**1**_, and ***A***_**2**_ are the parameters (vectors) used to estimate. It is noteworthy that all variables on the right hand of the models are one-period-lagged, to alleviate the endogeneity; 3,185 observations were used and 11,640 observations remained in the tests.

#### 3.3.2 Overidentification test

This section determines whether it is best to estimate panel data with fixed or random effects. Estimating with fixed effects requires the assumption that *ε*_*it*_ is irrelevant to *SA*_*it*_ and *lnPltcSbsd*_*it*_, given considering *FE*_*it*_ (fixed effects of corporation, time, and industry). Otherwise, estimating with random effects requires the assumption that given considering *FE*_*it*_ (fixed effects of corporation, time, and industry), the residual *ε*_*it*_ is still relevant to *SA*_*it*_ or *lnPltcSbsd*_*it*_, namely, some influencing factors are ignored in the model setting, but nonetheless exist in reality. Thus, the overidentification test (i.e., the Hausman test) was applied. According to the result, the Sargon-Hansen statistic χ^2^(34) = 549.716, and is statistically significant at the 1% significance level. Accordingly, the panel data were estimated with fixed effects.

#### 3.3.3 Mediation test

The mediation test was applied according to the three-step method of Baron and Kenny (1986) and Guo and Xu (2014) [62, 63]. The first step is to test model (1) mentioned in Section 3.3.1, and the models of the second and third steps are as follows:

$$lnPltcSbsd_{it}=\beta_{0}+\beta_{1}SA_{it}+B_{1}Control_{it}+B_{2}{FE}_{it}+\varepsilon_{it}$$ (2)

$$\ln\;Apply_{it}=c_{0}+c_{1}SA_{it}+C_{1}Control_{it}+C_{2}{FE}_{it}+\varepsilon_{it}$$ (3)

According to the three-step method of Baron and Kenny (1986) [62] and Guo and Xu (2014) [63], a mediation test is applied based on models (1), (2), and (3). Model (2) verifies whether financial constraints attract government subsidies. Considering *SA*_*it*_ is a negative index, a larger *SA*_*it*_ denotes a less severe financial constraint; a statistically significant and positive *β*_1_ indicates that less-severe-financial-constraint corporations tend to be subsidized more. Model (3) verifies whether financial constraints promote corporate innovation without considering government subsidies. Similar to model (2), a statistically significant and positive *c*_1_ indicates that less severely financially constrained corporations tend to innovate more.

Taking the results of these three models together, a mediation of government subsidies between financial constraints and innovation can be found with statistically significant *α*_2_ and *β*_1_. Moreover, given that *β*_1_ and *c*_1_ are statistically significant, model (1) helps to verify how government subsidies mediate between financial constraints and corporate innovation; that is, a government subsidy is a partial mediator if *α*_1_ is statistically significant, or a complete mediation.

## 4 Results and analysis

### 4.1 Results of the baseline model

**Fig 2:**
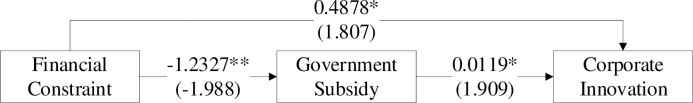
Main results of baseline model. [1] t statistics in parentheses. [2] *, p<0.1; **, p<0.05; ***, p<0.01.

The main results of the baseline models are shown in Table 3, where models (1)-(3) show the results of the linear models (the main results are shown in Fig 2), and models (4)-(6) show the results of non-linear models. In general, the majority of our hypotheses were supported.

**Table 3:** Main results of baseline model.

Model	(1)	(2)	(3)	(4)	(5)	(6)
Variable	*lnApply*_*it*_	*lnPltcSbsd*_*it*_	*lnApply*_*it*_	*lnApply*_*it*_	*lnPltcSbsd*_*it*_	*lnApply*_*it*_
*ApplyPerNtCpt*_*it*_	0.1503***	-0.0220	0.1507***	0.1496***	-0.0225	0.1499***
	(2.624)	(-0.277)	(2.631)	(2.616)	(-0.283)	(2.623)
*ApplyPerLbSlr*_*it*_	0.2173**	0.0185	0.2175**	0.2168**	0.0182	0.2170**
	(2.087)	(0.600)	(2.088)	(2.087)	(0.587)	(2.088)
*PltcCnct*_*it*_	-0.0379	-0.0226	-0.0384	-0.0398	-0.0241	-0.0403
	(-1.274)	(-0.321)	(-1.294)	(-1.342)	(-0.341)	(-1.361)
*SA*_*it*_	0.4919*	-1.2327**	0.4878*	3.2685***	0.8437	3.2335***
	(1.818)	(-1.988)	(1.807)	(2.863)	(0.289)	(2.842)
*SAsq*_*it*_				-0.0719**	-0.0538	-0.0711**
				(-2.481)	(-0.749)	(-2.462)
*lnPltcSbsd*_*it*_			0.0119*			0.0115*
			(1.909)			(1.846)
Controls	Yes	Yes	Yes	Yes	Yes	Yes
FE & Cons	Yes	Yes	Yes	Yes	Yes	Yes
N	11640	11640	11640	11640	11640	11640
*R* ^2^	0.270	0.172	0.270	0.271	0.172	0.271
Adj. *R*^2^	0.267	0.169	0.267	0.268	0.169	0.268

Note: [1]

*, **, and *** denote significance at the 10%, 5%, and 1% significance levels. [2] t values are reported in parentheses. [3] Columns of Controls and FE & Cons denotes whether control variables, fixed effects, and constant are included. [4] Independent variables and control variables are lagged one year. [5] Robust standard error is applied. [6] *R*^2^ denotes R square within groups, Adj. *R*^2^ denotes R square within groups adjusted by the number of variables.

The main results of the linear models (1)-(3) are shown in Fig 2. Regarding the potential non-linear influence of financial constraints, insufficient evidence was found according to the results of the linear models (4)-(6). Thus, the conclusions are mainly drawn upon the linear models, and the results of non-linear models only play a robustness-test role.

According to the results of model (2), H1 was supported. The influence of financial constraints on government subsidies is negative and statistically significant at the 5% significance level, indicating that corporations with less severe financial constraints (*SA*_*it*_ is a negative index) receive fewer government subsidies. Meanwhile, the coefficients of *PltcCnct*_*it*_, *ApplyPerNtCpt*_*it*_, and *ApplyPerLbSlr*_*it*_ in model (2) are not statistically significant, indicating that the main factor attracting government subsidies is financial constraints. These results suggest that H1 is supported.

According to the results of model (3), H2 was supported, which is consistent with the results of Carboni (2017) [17] and Mateut (2018) [20]. The coefficient of *lnPltcSbsd*_*it*_ on *lnApply*_*it*_ in model (3) is 0.0119 and statistically significant at the 10% significance level, indicating that corporations receiving more subsidies innovate more.

According to the results of model (3), financial constraints suppress corporate innovation. The influence of *SA*_*it*_ on *lnApply*_*it*_ is statistically significant and positive at the 10% significance level, indicating that corporations with less severe financial constraints innovate more. Thus, H3 was supported, which is consistent with Mateut (2018) [20].

As for the non-linear relationship between financial constraints and corporate innovation, our proof might be insufficient to conclude an inverted-U relationship. Model (5) considers the impact of square-form *SA*_*it*_ (namely *SAsq*_*it*_) on government subsidies, and the result is not statistically significant. That is, no evidence of a non-linear relationship between government subsidies and financial constraints is found. According to the results of model (6), the influence of *SAsq*_*it*_ on *lnApply*_*it*_ is negative and statistically significant at the 1% significance level. However, considering the distribution of *SA*_*it*_ (whose maximum value in our sample is no more than 11), although the proof of an inverted U-shaped relationship (whose peak is located at 23.07) between financial constraints and corporate innovation can be found, all A-list corporations are located on the left subset of the inverted U-shape. Thus, considering the maximum *SA*_*it*_ being no more than 11 and the peak located at 23.07, our observation data range is only a left subset of the inverted-U shape, and cannot conclude the existence of an inverted U-shaped relationship [64].

### 4.2 Results of mediation test

The main results of the mediation tests are shown in Table 3. Models (1)-(3) construct a set of tests, and models (4)-(6) construct another set that considers the nonlinear relationship of financial constraints (*SAsq*_*it*_). Generally speaking, the mediating mechanism (namely H4) was supported, as H1, H2, and H3 were supported, as mentioned in Section 4.1.

Government subsidies partially mediate the relationship between financial constraints and corporate innovation. On the one hand, subsidies mediate between financial constraints and innovation, according to the statistically significant coefficients of *SA*_*it*_ in model (2) and *lnPltcSbsd*_*it*_ in model (3). On the other hand, according to model (3), the coefficient of *SA*_*it*_ is statistically significant, indicating that government subsidies partially mediate between financial constraints and corporate innovation. Moreover, according to the statistically significant coefficient of *SA*_*it*_ in model (1), the total effect of financial constraint on corporate innovation is 0.4919, which indicates that financial constraint has a negative impact on corporate innovation (*SA*_*it*_ negatively indicates financial constraints). Meanwhile, according to the statistically significant coefficients of *SA*_*it*_ in model (3), the direct effect of financial constraint on corporate innovation is 0.4878, which indicates that the total effect is greater than the direct effect by 0.0041 (0.4919−0.4878).

Furthermore, the mediating effect of government subsidies is competing, namely government subsidies targeting at bailing out financial constraints are able to set off the negative effect of financial constraints on innovation. According to the significant coefficients of *SA*_*it*_ in model (1) and *lnPltcSbsd*_*it*_ in model (3) which are respectively −1.2327 and 0.0119, the indirect effect of financial constraints on corporate innovation via government subsidies is −0.0147 (−1.2327×0.0119). This negative indirect effect indicates a competing mediation that the bailing-out government subsidies reduce the negative impact of financial constraints on corporate innovation. Moreover, the ratio of indirect effect to direct effect is around 3.01% (0.0147÷0.4878), which is the approximate amount of how much bailing-out subsidies partially and competitively mediate between financial constraints and innovation.

When considering the potential inverted U-shaped influence of financial constraints, the conclusion remains mostly robust. On the one hand, financial constraints attract government subsidies according to model (2), while no evidence of an inverted U-shaped influence of financial constraints on government subsidies is found according to model (5). These results indicate the robustness of H1. On the other hand, based on models (4) and (6), the influences of *SAsq*_*it*_ on *lnApply*_it_ are both positive and statistically significant and the results of *SA*_*it*_ and *lnPltcSbsd*_*it*_ remain robust, indicating the robustness of H2. Together, these two results provide evidence of the robustness of H4.

### 4.3 Results of moderation test

According to the most recent research of Acebo and Miguel-Dávila (2020) [14], the possible moderating effect of financial constraints on how government subsidies influence corporate innovation is considered by introducing an interacting term of subsidy and financial constraint, which is denoted as *PSSA*_*it*_ = *lnPltcSbsd*_it_*SA_*it*_.

The main results are shown in Table 4, but there is insufficient evidence to support the finding of Acebo and Miguel-Dávila (2020) that there is a moderating effect of government subsidies and financial constraints on corporate innovation [14]. According to model (3), although the coefficient of *PSSA*_*it*_ is statistically significant, the coefficients of the main effects (*SA*_*it*_ and *lnPltcSbsd*_*it*_) are not statistically significant. Thus, we cannot conclude a moderating effect between subsidies and financial constraints in a universal context.

**Table 4:** Main results of moderating model.

Model	(1)	(2)	(3)	(4)
Variable	*lnApply*_*it*_	*lnApply*_*it*_	*lnApply*_*it*_	*lnApply*_*it*_
*SA*_*it*_	0.4919*	0.4878*	0.1080	2.8377**
	(1.818)	(1.807)	(0.319)	(2.452)
*lnPltcSbsd*_*it*_		0.0119*	-0.0227	-0.0223
		(1.909)	(-1.177)	(-1.161)
*lnPSSA*_*it*_			0.0091*	0.0089*
			(1.950)	(1.915)
*SAsq*_*it*_				-0.0705**
				(-2.450)
Controls	Yes	Yes	Yes	Yes
FE & Cons	Yes	Yes	Yes	Yes
*N*	11640	11640	11640	11640
*R* ^2^	0.270	0.270	0.271	0.272
adj. *R*^2^	0.267	0.267	0.268	0.269

Note: [1]

*, **, and *** denote significance at the 10%, 5%, and 1% significance levels. [2] t values are reported in parentheses. [3] Columns of Controls and FE & Cons denotes whether control variables, fixed effects, and constant are included. [4] Independent variables and control variables are lagged one year. [5] Robust standard error is applied. [6] *R*^2^ denotes R square within groups, Adj. *R*^2^ denotes R square within groups adjusted by the number of variables.

## 5 Robustness test

### 5.1 Subsamples of different innovation strategies

As mentioned, in China, patents are sorted into three categories: invention, utility model, and industrial design. Invention patents are granted for revolutionary solutions, utility patents are granted for new and practical solutions related to a physical structure, and design patents are granted for a new appearance [65].

According to mainstream research in China [66], corporate innovation strategies are divided into two categories: exploratory and exploitative innovation. Exploratory innovation mainly strives to combine knowledge elements in a novel manner to transform a business, while exploitative innovation reconfigures existing combinations so that they can be put to new uses and applications.

The number of invention patents in logarithm is used to measure the exploratory innovation strategy (*ln IApply*_*it*_) because the input and difficulty of invention are larger than those of the other two. The gross number of utility model and industrial design patents in logarithm is used to measure the exploitative innovation strategy (*ln SApply*_*it*_); the number of utility models and industrial designs are also considered, denoted as *ln UApply*_*it*_ and *ln DApply*_*it*_.

The results of the subsamples divided by innovation strategy are shown in Tables 5 and 6. Table 5 shows the results of the subsample for the exploratory innovation strategy, while Table 6 shows the results of the subsample for the exploitative innovation strategy. Generally speaking, our major conclusions remain robust, that is, financial constraints suppress corporate exploratory innovation, while government subsidies offset this suppression by partial and competitive mediation.

**Table 5:** Subsample results of exploratory innovation strategy.

Model	(1)	(2)	(3)	(4)	(5)	(6)
Variable	*lnIApply*_*it*_	*lnPltcSbsd*_*it*_	*lnIApply*_*it*_	*lnIApply*_*it*_	*lnPltcSbsd*_*it*_	*lnIApply*_*it*_
*IApplyPerNtCpt*_*it*_	0.1962*	0.0240	0.1968*	0.0246	0.0246	0.1975**
	(1.943)	(0.185)	(1.954)	(0.190)	(0.190)	(1.970)
*IApplyPerLbSlr*_*it*_	0.5268**	0.0340	0.5285**	0.0331	0.0331	0.5274**
	(1.982)	(0.326)	(1.984)	(0.316)	(0.316)	(1.983)
*PltcCnct*_*it*_	-0.0435	-0.0224	-0.0443	-0.0238	-0.0238	-0.0461
	(-1.422)	(-0.318)	(-1.445)	(-0.338)	(-0.338)	(-1.507)
*SA*_*it*_	0.5110*	-1.2276**	0.5060*	0.8481	0.8481	3.1323***
	(1.799)	(-1.979)	(1.786)	(0.291)	(0.291)	(2.641)
*SAsq*_*it*_				-0.0538	-0.0538	-0.0680**
				(-0.750)	(-0.750)	(-2.294)
*lnPltcSbsd*_*it*_			0.0148**			0.0144**
			(2.071)			(2.013)
Controls	Yes	Yes	Yes	Yes	Yes	Yes
FE & Cons	Yes	Yes	Yes	Yes	Yes	Yes
N	11640	11640	11640	11640	11640	11640
*R* ^2^	0.268	0.172	0.268	0.269	0.172	0.269
Adj. *R*^2^	0.265	0.169	0.265	0.266	0.169	0.266

Note: [1]

*, **, and *** denote significance at the 10%, 5%, and 1% significance levels. [2] t values are reported in parentheses. [3] Columns of Controls and FE & Cons denotes whether control variables, fixed effects, and constant are included. [4] Independent variables and control variables are lagged one year. [5] Robust standard error is applied. [6] *R*^2^ denotes R square within groups, Adj. *R*^2^ denotes R square within groups adjusted by the number of variables.

**Table 6:** Subsample results of exploitative innovation strategy.

Model	(1)	(2)	(3)	(4)	(5)	(6)	(7)	(8)
Variables	*lnPltcSbsd*_*it*_	*lnSApply*_*it*_	*lnUApply*_*it*_	*lnDApply*_*it*_	*lnPltcSbsd*_*it*_	*lnSApply*_*it*_	*lnUApply*_*it*_	*lnDApply*_*it*_
*SA*_*it*_	-1.2327**	0.6937**	0.7428***	0.3510	0.8437	3.3765***	3.6141***	1.6164
	(-1.988)	(2.443)	(2.612)	(1.378)	(0.289)	(2.644)	(2.773)	(1.511)
*SAsq*_*it*_					-0.0538	-0.0695**	-0.0744**	-0.0328
					(-0.749)	(-2.145)	(-2.249)	(-1.174)
*lnPltcSbsd*_*it*_		0.0095	0.0114	0.0067		0.0091	0.0110	0.0066
		(1.348)	(1.580)	(1.132)		(1.295)	(1.522)	(1.100)
Controls	Yes	Yes	Yes	Yes	Yes	Yes	Yes	Yes
FE & Cons	Yes	Yes	Yes	Yes	Yes	Yes	Yes	Yes
N	11640	11640	11640	11640	11640	11640	11640	11640
R2	0.172	0.172	0.232	0.054	0.172	0.204	0.233	0.054
Adj. R2	0.169	0.169	0.229	0.050	0.169	0.200	0.230	0.050

Note: [1]

*, **, and *** denote significance at the 10%, 5%, and 1% significance levels. [2] t values are reported in parentheses. [3] Columns of Controls and FE & Cons denotes whether control variables, fixed effects, and constant are included. [4] Independent variables and control variables are lagged one year. [5] Robust standard error is applied. [6] *R*^2^ denotes R square within groups, Adj. *R*^2^ denotes R square within groups adjusted by the number of variables.

#### 5.1.1 Subsamples of exploratory innovation strategy

**Fig 3:**
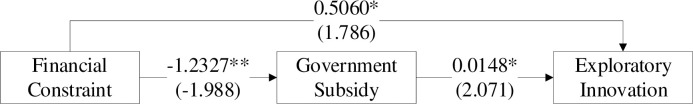
Main results of subsample with an exploratory innovation strategy. [1] t statistics in parentheses. [2] *, p<0.1; **, p<0.05; ***, p<0.01.

Table 5 shows the results of the subsample with exploratory innovation strategy and the main results are shown in Fig 3.

On the one hand, financial constraints suppress corporate exploratory innovation. In Table 5, the influence between *SA*_*it*_ and *lnIApply*_*it*_ in models (1) and (3) are statistically significant and positive, indicating that corporations with less severe financial constraints have more exploratory innovation. Specifically, according to model (6), even considering the possible non-linear influence of financial constraints, the conclusion that financial constraints suppress corporate exploratory innovation remains, because the distribution of China A-list corporations’ SA index is located on the left of the peak.

On the other hand, government subsidies set off suppression of financial constraints on innovation by partial and competitive mediation. According to model (1), the total effect of financial constraints on innovation is 0.5110. according to model (3), the direct effect is 0.5060, and the indirect effect via government subsidies is −0.0182 (−1.2276×0.0148). Thus, the indirect effect indicates that government subsidies mediate the impact of financial constraints on innovation, the direct effect indicates that subsidies are partial mediator, and the negative indirect effect indicates the mediation is competitive. Meanwhile, considering the possible inverted U influence between financial constraints and exploratory innovation, according to model (6), subsidy promotes corporate innovation with an elasticity of 0.0144 with a 5% significance level. This result increases the credibility that government subsidies set off suppression by partial and competitive mediation.

#### 5.1.2 Subsamples of exploitative innovation strategy

Table 6 shows the results of the subsample with an exploratory innovation strategy. Generally speaking, some conclusions remain robust, which means that financial constraints suppress corporate exploratory innovation. Interestingly, financial constraints suppress innovation of utility but do not suppress innovation of industrial design. Moreover, evidence shows that government subsidies do not promote exploitative innovation.

On the one hand, financial constraints suppress corporate exploitative innovation (mainly utility model patents). The influence of *SA*_*it*_ on *lnSApply*_*it*_ in model (2) is positive and statistically significant at the 5% significance level, indicating that corporations with less severe financial constraints exploitatively innovate more. Meanwhile, according to model (6), considering the possible inverted-U relationship of financial constraints, the conclusion that financial constraints suppress corporate exploitative innovation remains, because the distribution of China A-list corporations’ SA index is located on the left of the peak.

On the other hand, there is no evidence supporting the promotion of government subsidies on exploitative innovation. No coefficient of government subsidy is statistically significant under the condition of exploitative innovation strategy, indicating that government subsidies affect exploratory innovation more and that they are efficient.

Furthermore, delving deeper into the specific patent types of exploitative strategy, the difference between the utility model and industrial design can be found. According to the results of models (3), (4), (7), and (8) in Table 6, there is no evidence of industrial design patents being suppressed by financial constraints, which is different from the utility model. These results indicate a structural difference in financial constraints on corporate innovation types and provide the rationale for promoting high-quality corporate innovation by alleviating financial constraints.

#### 5.1.3 Moderation tests on different innovation strategies

Moderation tests are also applied to subsamples divided by innovation strategies, and the main results are presented in Table 7. Models (1) and (2) show the results of the moderation tests on the subsample with an exploratory innovation strategy. Models (3)-(8) show the results of moderation tests on the subsample with exploitative innovation strategy, models (3) and (4) show those of exploitative innovation, models (5) and (6) show those of the utility model, and models (7) and (8) show those of industrial design.

**Table 7:** Main results of moderation tests on different innovation strategies.

Model	(1)	(2)	(3)	(4)	(5)	(6)	(7)	(8)
Strategy	Exploratory Innovation		Exploitative Innovation					
Variable	lnIApply_it_		lnSApply_it_		lnUApply_it_		lnDApply_it_	
*SA*_*it*_	0.0930	2.7007**	0.2252	2.8858**	0.3331	3.1867**	-0.3463	0.8780
	(0.269)	(2.246)	(0.603)	(2.230)	(0.915)	(2.414)	(-1.159)	(0.818)
*SAsq*_*it*_		-0.0673**		-0.0687**		-0.0737**		-0.0316
		(-2.282)		(-2.131)		(-2.235)		(-1.144)
*lnPltcSbsd*_*it*_	-0.0228	-0.0224	-0.0331	-0.0328	-0.0259	-0.0255	-0.0567***	-0.0566***
	(-1.103)	(-1.083)	(-1.492)	(-1.482)	(-1.176)	(-1.162)	(-3.080)	(-3.073)
*PSSA*_*it*_	0.0099**	0.0097**	0.0112**	0.0110**	0.0098*	0.0096*	0.0167***	0.0166***
	(2.043)	(1.998)	(2.075)	(2.054)	(1.839)	(1.811)	(3.768)	(3.753)
Controls	Yes	Yes	Yes	Yes	Yes	Yes	Yes	Yes
FE & Cons	Yes	Yes	Yes	Yes	Yes	Yes	Yes	Yes
N	11640	11640	11640	11640	11640	11640	11640	11640
*R* ^2^	0.269	0.269	0.203	0.204	0.233	0.234	0.055	0.056
adj. *R*^2^	0.266	0.266	0.200	0.201	0.230	0.230	0.051	0.052

Note: [1]

*, **, and *** denote significance at the 10%, 5%, and 1% significance levels. [2] t values are reported in parentheses. [3] Columns of Controls and FE & Cons denotes whether control variables, fixed effects, and constant are included. [4] Independent variables and control variables are lagged one year. [5] Robust standard error is applied. [6] *R*^2^ denotes R square within groups, Adj. *R*^2^ denotes R square within groups adjusted by the number of variables.

In summary, there is not sufficient evidence to support the finding of Acebo and Miguel-Davila (2020) that there is a moderating relationship between government subsidy and financial constraint [14]. The coefficients of *PSSA*_*it*_ are statistically significant and positive in every model, which indicates that corporations with less severe financial constraints and more subsidies tend to innovate more. However, the main effects in the subsample-moderation-test models are not all statistically significant; thus, we cannot conclude the existence of a moderating relationship between government subsidies and financial constraints.

## 5.2 Different estimation

### 5.2.1 Time-invariant fixed effects

The above results are based on the fixed-effect estimation and consider three fixed effects of individual, time, and industry. However, considering the scale of missing data, time-invariant fixed effects might make a difference. Thus, an alternative fixed-effect estimation is applied. The model setting is similar to the above models, but the estimation is different: only individual fixed effects and industrial fixed effects are considered.

According to the results shown in Table 8, the main conclusions remain robust. That is, while the current financial constraints of Chinese-listed corporations suppress their innovation, government subsidies promote corporate innovation. However, with the time-invariant fixed-effect estimation, the adjusted *R*^2^ of model (2) decreases sharply (around 94.3%) and the mediation effect of government subsidy becomes statistically insignificant, which suggests the existence of a time-varying effect, which might be caused by the yearly changes in the global and financial context.

**Table 8:** Main results of time-invariant fixed effects.

Model	(1)	(2)	(3)	(4)	(5)	(6)
Variable	*lnApply*_*it*_	*lnPltcSbsd*_*it*_	*lnApply*_*it*_	*lnApply*_*it*_	*lnPltcSbsd*_*it*_	*lnApply*_*it*_
*ApplyPerNtCpt*_*it*_	0.1542***	-0.0172	0.1545***	0.1539***	-0.0175	0.1543***
	(2.702)	(-0.203)	(2.711)	(2.703)	(-0.206)	(2.711)
*ApplyPerLbSlr*_*it*_	0.2181**	0.0321	0.2182**	0.2178**	0.0317	0.2179**
	(2.082)	(0.938)	(2.083)	(2.083)	(0.923)	(2.085)
*PltcCnct*_*it*_	-0.0292	-0.0234	-0.0301	-0.0305	-0.0252	-0.0314
	(-1.000)	(-0.325)	(-1.032)	(-1.049)	(-0.351)	(-1.079)
*SA*_*it*_	0.4635*	-0.7951	0.4559*	2.8515**	2.4882	2.7990**
	(1.717)	(-1.255)	(1.693)	(2.510)	(0.836)	(2.472)
*SAsq*_*it*_				-0.0619**	-0.0852	-0.0608**
				(-2.143)	(-1.149)	(-2.110)
*lnPltcSbsd*_*it*_			0.0123**			0.0118*
			(2.020)			(1.947)
Controls	Yes	Yes	Yes	Yes	Yes	Yes
FE & Cons	Yes	Yes	Yes	Yes	Yes	Yes
N	11640	11640	11640	11640	11640	11640
*R* ^2^	0.264	0.090	0.264	0.265	0.090	0.265
Adj. *R*^2^	0.262	0.087	0.262	0.262	0.087	0.263

Note: [1]

*, **, and *** denote significance at the 10%, 5%, and 1% significance levels. [2] t values are reported in parentheses. [3] Columns of Controls and FE & Cons denotes whether control variables, fixed effects, and constant are included. [4] Independent variables and control variables are lagged one year. [5] Robust standard error is applied. [6] *R*^2^ denotes R square within groups, Adj. *R*^2^ denotes R square within groups adjusted by the number of variables.

#### 5.2.2 Random effects

Along with the time-invariant fixed effects, random effects caused by factors that have hitherto been ignored might also make a difference. Thus, random-effect estimation was also applied, and the other settings remained the same.

According to the results shown in Table 9, the main conclusions remain robust. That is, financial constraints draw government subsidies, which promote corporate innovation; during this process, they play the role of mediator.

**Table 9:** Main results of random effects.

Model	(1)	(2)	(3)	(4)	(5)	(6)
Variable	*lnApply*_*it*_	*lnPltcSbsd*_*it*_	*lnApply*_*it*_	*lnApply*_*it*_	*lnPltcSbsd*_*it*_	*lnApply*_*it*_
*ApplyPerNtCpt*_*it*_	0.0419	0.0066	0.0412	0.0419	0.0074	0.0411
	(1.283)	(0.942)	(1.275)	(1.283)	(1.053)	(1.272)
*ApplyPerLbSlr*_*it*_	0.3626**	0.0803*	0.3682**	0.3636**	0.0794*	0.3697**
	(2.350)	(1.821)	(2.362)	(2.351)	(1.803)	(2.363)
*PltcCnct*_*it*_	-0.0412	0.0488	-0.0471*	-0.0414	0.0482	-0.0474*
	(-1.519)	(0.843)	(-1.739)	(-1.529)	(0.831)	(-1.749)
*SA*_*it*_	0.1701	-0.7259*	0.1434	0.4917	1.2492	0.2528
	(0.825)	(-1.913)	(0.704)	(0.543)	(0.734)	(0.283)
*SAsq*_*it*_				-0.0078	-0.0463	-0.0027
				(-0.362)	(-1.244)	(-0.126)
*lnPltcSbsd*_*it*_			0.0468***			0.0471***
			(7.071)			(7.105)
Controls	Yes	Yes	Yes	Yes	Yes	Yes
Cons	Yes	Yes	Yes	Yes	Yes	Yes
N	11640	11640	11640	11640	11640	116401
*R*^2^ within	0.247	0.086	0.245	0.247	0.087	0.245
*R*^2^ between	0.166	0.193	0.192	0.166	0.193	0.192
*R*^2^ overall	0.230	0.187	0.250	0.230	0.188	0.250

Note: [1]

*, **, and *** denote significance at the 10%, 5%, and 1% significance levels. [2] t values are reported in parentheses. [3] Columns of Controls and FE & Cons denotes whether control variables, fixed effects, and constant are included. [4] Independent variables and control variables are lagged one year. [5] Robust standard error is applied.

#### 5.2.3 Dynamic model

The potential dynamic panel bias is discussed in this section. Given the assumption that the variables are not autocorrelated, the fixed-effect estimation provides consistent estimators. However, there may be a self-momentum of corporate innovation, and government subsidies might last for several years. Thus, it is necessary to consider the dynamic panel bias.

To achieve this, two approaches were applied. One was based on the baseline fixed-effect models with up-to-three-period lagging of both explained and explanatory variables introduced to capture the potential autocorrelation. For the second, dynamic panel data models with system GMM estimation are applied following Arellano and Bover (1995) and Blundell and Bond (1998) [67, 68]. In our dynamic model, up-to-three-period lagging of both explained and explanatory variables were used as bases for “GMM-style” instrument sets.

**Fig 4:**
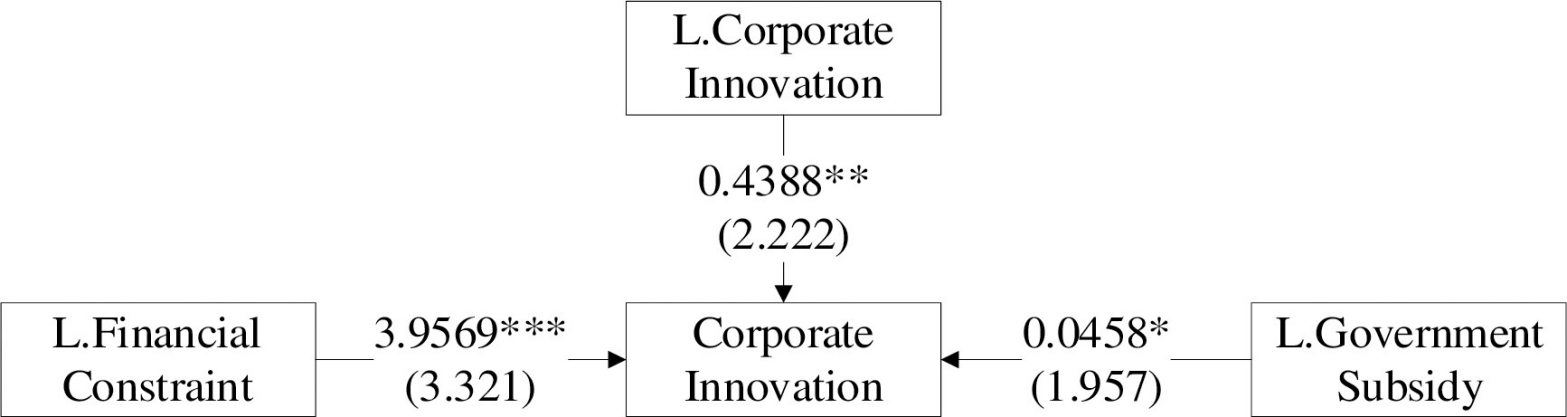
Main results of model (4) in Table 10. [1] t statistics in parentheses. [2] *, p<0.1; **, p<0.05; ***, p<0.01.

The results of the dynamic approaches are shown in Table 10, and the main results are shown in Fig 4, and generally support our main conclusions. Models (1)-(3) show the results of the fixed-effect estimation. According to the statistically significant coefficients of each lagging *lnApply*_*it*_, there might be a bias in the dynamic panel when considering the autocorrelation of corporate innovation. Thus, the results of fixed-effect estimation by simply introducing lagging variables might be unreliable. Models (4)-(6) show the results of the system-GMM estimation. According to the autocorrelation tests that only AR(1) in model (6) is statistically significant, the assumption that errors are not autocorrelated stands; thus, the results of autocorrelation tests indicate that it is suitable to apply the system-GMM estimation.

**Table 10:** Main results of dynamic model.

	(1)	(2)	(3)	(4)	(5)	(6)
Estimation	Fixed-effect			System GMM		
Variables	*lnApply*_*it*_					
*l*. *lnApply*_*it*_	0.2315***	0.1811***	0.1497***	0.4388**	0.4238***	0.1589
	(15.203)	(10.807)	(8.180)	(2.222)	(3.760)	(1.325)
*l*2. *lnApply*_*it*_		0.0511***	0.0374**		0.0788	0.1611***
		(3.765)	(2.411)		(1.181)	(4.120)
*l*3. *lnApply*_*it*_			-0.0444***			0.1474***
			(-3.273)			(2.600)
*l*. *SA*_*it*_	0.3973*	0.0591	0.1173	3.9569***	2.0105**	2.6419**
	(1.760)	(0.212)	(0.350)	(3.321)	(2.047)	(2.482)
*l*2. *SA*_*it*_		0.0492	0.0570		0.2220	0.5881**
		(1.115)	(1.187)		(0.755)	(2.185)
*l*3. *SA*_*it*_			-0.0100			-0.4157**
			(-0.263)			(-2.542)
*l*. *lnPltcSbsd*_*it*_	0.0074	-0.0015	0.0086	0.0458*	0.0315	0.0370
	(1.272)	(-0.207)	(0.820)	(1.957)	(1.337)	(1.452)
*l*2. *lnPltcSbsd*_*it*_		0.0093	0.0078		0.0621**	0.0474**
		(1.506)	(0.909)		(2.338)	(2.313)
*l*3. *lnPltcSbsd*_*it*_			0.0028			0.0347
			(0.429)			(1.166)
Controls, FE & Cons	Yes	Yes	Yes	Yes	Yes	Yes
*N*	11640	9430	7645	11640	9430	7645
*R* ^2^	0.304	0.249	0.193			
adj. *R*^2^	0.301	0.245	0.188			
AR(1)				-1.38	-2.00	-4.79***
AR(2)				-1.33	-0.97	-1.10
Sargan				2362.95	3339.43	3056.19
Hansen				487.69	610.21	555.94

Note: [1]

*, **, and *** denote significance at the 10%, 5%, and 1% significance levels. [2] t values are reported in parentheses of fixed-effect estimation, z values are reported in parentheses of system GMM estimation. [3] Controls, FE & Cons denotes whether control variables, fixed effects, and constant are included. [4] Robust standard error is applied in fixed-effect estimation, robust estimator of the covariance matrix of the parameter estimates is applied in system GMM estimation. [5] *R*^2^ denotes R square within groups, adj. *R*^2^ denotes R square within groups adjusted by the number of variables.

The main results of system-GMM estimation are as follows. First, corporate innovation is autocorrelated, considering the statistically significant coefficients of *l*. *lnApply*_*it*_ in model (4), *l*. *lnApply*_*it*_ in model (5), and *l*2. *lnApply*_*it*_ and *l*3. *lnApply*_*it*_ in model (6). Second, less severe financial constraints promote innovation, according to the statistically significant coefficients of *l*. *SA*_*it*_ in models (4)-(5) and *l*2. *SA*_*it*_ in model (6). Finally, government subsidies promote corporate innovation, as indicated by the statistically significant coefficients of *l*. *lnPltcSbsd*_*it*_ in models (4) and *l*2. *lnPltcSbsd*_*it*_ in models (5) and (6).

## 6 Conclusion

To grasp the process of government subsidy allocation [1–3] and to determine the effectiveness of subsidies on promoting corporate innovation and whether they crowd out financial capital [14], we investigated the relationships between financial constraint, government subsidies, and corporate innovation.

To do so, we introduced innovative ability, political connections, and financial constraints as potential targets of government subsidies. Subsequently, we considered corporate innovation to test the effectiveness of government subsidies. We constructed a semi-logarithmic fixed-effect panel regression, mediation effect test, and moderation effect test based on the unbalanced panel data of Chinese-listed companies from 2007 to 2017. In addition, we applied several robustness tests, including different innovation strategies and estimation methods.

By doing so, we found:

1. Financial constraints suppress corporate innovation, consistent with Lin and Liu (2017) [58] and Mateut (2018) [20]. This result also supports the argument of Giebel and Kraft (2020) that corporate innovation reacts sensitively to financing, and funding shortages lead to a higher probability of less innovation [33].
2. Financial constraints attract government subsidies. This result reveals one of the targeting strategies of government subsidies in China, which is to bail out financially constrained corporations. This result contrasts with that of Silva and Carreira (2017) who argue that financial constraints are not taken into consideration during subsidy allocation [8]. One possible reason might be the different nations of the samples; our result is drawn based on Chinese corporations, while Silva and Carreira (2017) draw theirs based on Portuguese corporations. Another possible reason might be the different time range of the samples. Moreover, the bail-out target of government subsidies highlights the probable problem of endogeneity in the existing research on the relationship between subsidies and financial constraints [9, 10].
3. Government subsidies promote corporate innovation, consistent with the finding of Huergo and Trenado (2016) [35], Carboni (2017) [17], Mateut (2018) [20], and Ivus and Jose (2021) [32]. However, this result somewhat contradicts that of Acebo and Miguel-Dávila (2020) who found that subsidies alone do not increase investment in corporate innovation [14]. The reason for this may be the different measures of corporate innovation. We measure innovation using patents emphasizing the output of corporate innovation, while Acebo and Miguel-Dávila (2020) measure innovation using R&D investment considering the input [14]. Thus, these contrasting conclusions indicate that subsidies might promote corporate innovation output by lifting innovative efficiency instead of innovative input [69, 70], which deserves further research.
4. Government subsidies mediate between financial constraints and corporate innovation, where the mediation is partial and competitive. That is, corporations with more severe financial constraints tend to generate less innovation, while government subsidies tend to be given to these more severely financially constrained corporations; and more subsidies promote more innovation outcomes. Thus, these constrained corporations with more subsidies generate more innovation outcomes than those with fewer subsidies. This mitigation might be due to the fact that subsidies alleviate underinvestment in corporate innovation [58] and signal the innovative natures of subsidized corporations [71]. This result is consistent with that of Carboni (2017) [17] and Mateut (2018) [20] and positively evaluates the effectiveness of government subsidies. That is, government subsidies promote corporate innovation and thus create long-term social benefits beyond merely bailing out financially constrained corporations.
5. In the context of different innovation strategies, the relationships among subsidies, financial constraints, and innovation differ. As for the exploratory strategy, financial constraints suppress exploratory innovation, and subsidies partially and competitively mediate this process; that is, the bail-out subsidy indeed sets off the innovation suppression of financial constraints. As for the exploitative strategy, financial constraints suppress utility innovation, while no evidence is found in the other relationships. These results support the argument of Hottenrott and Lopes-Bento (2017) who found different influences of subsidies on different innovation approaches [72]. Our results also support Giebel and Kraft (2020) that different types of innovation are affected by financial constraints in different ways [33].

Based on the conclusions, we contribute to the fields of public finance, corporate finance, and corporate innovation.

First, we justified the government subsidy targeting strategy of bailing out financially constrained corporations. Our findings point to the endogenous question in research relating to public fund allocation [9, 10]. Meanwhile, our findings absolve the Chinese government against accusations of unfair competition.

Second, we verified the effectiveness of government subsidies on corporate innovation promotion. We investigated one step further, mainly by verifying the positive effect of government subsidies on innovation outcomes, compared with the related literature, such as Dai and Cheng (2015) who find that subsidies promote innovation investment [38], and Huergo and Trenado (2016) [35] and Carboni (2017) [17] who find that subsidies incentivize innovation. Therefore, we justified government intervention in China and recommended a probable future research agenda for investigating the efficiency-lifting mechanism of government subsidies. What’s more, we contributed to the debate on whether government subsidies crowd out corporate innovation [7].

Third, we supported the argument that financial constraints suppress corporate innovation. This finding supports the classical theory that financial constraints hinder innovation [18, 20]. Considering the current financial-suppression conditions in China and taking innovation-driven developing targets into account, we recommended to either reform the financial market from the monetary side or to optimize subsidy allocation from the public finance side.

Finally, by dividing innovation into exploratory and exploitative strategies, we tested the efficacy of subsidies under different conditions following the arguments of Hottenrott and Lopes-Bento (2017), especially in transitioning and developing economies [72]. Our findings not only theoretically supported the existing research [33], but also justified government intervention in China. We found that different types of innovation are generally suppressed by financial constraints, while subsidies offset the suppression of exploratory ones. Considering these structural differences in subsidy promotion for different types of innovation, there is a rationale to subsidize financially constrained corporations. The expected benefit is more exploratory innovation, which is urgently needed in China to overcome the middle-income trap.
